# Mycotic Infections Acquired outside Areas of Known Endemicity, United States

**DOI:** 10.3201/eid2111.141950

**Published:** 2015-11

**Authors:** Kaitlin Benedict, George R. Thompson, Stan Deresinski, Tom Chiller

**Affiliations:** Centers for Disease Control and Prevention, Atlanta, Georgia, USA (K. Benedict, T. Chiller);; University of California Davis Medical Center, Davis, California, USA (G.R. Thompson III);; University of California, Davis (G.R. Thompson III);; Stanford University, Stanford, California, USA (S. Deresinski)

**Keywords:** lung diseases, mycoses, fungal, fungi, blastomycosis, coccidioidomycosis, histoplasmosis, endemicity, United States

## Abstract

In the United States, endemic mycoses—blastomycosis, coccidioidomycosis, and histoplasmosis—pose considerable clinical and public health challenges. Although the causative fungi typically exist within broadly defined geographic areas or ecologic niches, some evidence suggests that cases have occurred in humans and animals not exposed to these areas. We describe cases acquired outside regions of traditionally defined endemicity. These patients often have severe disease, but diagnosis may be delayed because of a low index of suspicion for mycotic disease, and many more cases probably go entirely undetected. Increased awareness of these diseases, with a specific focus on their potential occurrence in unusual areas, is needed. Continued interdisciplinary efforts to reevaluate and better describe areas of true endemicity are warranted, along with a more nuanced view of the notion of endemicity. The term “nonendemic” should be used with care; mycoses in such regions might more accurately be considered “not known to be endemic.”

## Abstract 2

ncreased awareness that mycoses can be acquired in unusual geographic locations is needed to promote early diagnosis and treatment.

Invasive fungal diseases are a growing public health problem. The endemic mycoses found in North America, namely, blastomycosis, coccidioidomycosis, and histoplasmosis, are caused by thermally dimorphic fungi and can infect immunocompetent or immunocompromised hosts, often resulting in severe illness and death (*1*). Infection is typically acquired via inhalation of fungal spores and usually results in a respiratory illness, although the clinical spectrum can range from asymptomatic to life-threatening disseminated disease (*1*–*4*). Most infections with *Blastomyces*, *Coccidioides*, and *Histoplasma* spp. occur sporadically in areas to which these fungi are geographically restricted, also referred to as mycosis-endemic areas (*1*). Cases outside these regions often result from travel, infection reactivation, latent infection in immunosuppressed hosts, or (less commonly) exposure to fomites from mycosis-endemic areas (*1*,*3*). However, a growing body of evidence suggests that some cases occur in patients with no known exposures to areas in which these diseases are most common. Because prevention of these infections is challenging, increased awareness that they can be acquired in unusual geographic locations is needed to promote early diagnosis and treatment.

## Search Strategy and Selection Criteria

To identify infections outside the known mycosis-endemic areas in the United States, we searched PubMed and Google Scholar without date or language restrictions by using combinations of the following terms: “non-endemic,” “outside endemic area,” “blastomycosis,” “histoplasmosis,” “coccidioidomycosis,” and “fungal infection.” We included publications in which the authors describe infections believed to be acquired from the local environment outside the traditionally defined mycosis-endemic areas in the United States. We excluded cases consistent with fomite transmission. We also reviewed relevant references in selected articles.

### Blastomycosis

Blastomycosis is considered endemic to the south-central, southeastern, and midwestern US states, particularly those bordering the Ohio and Mississippi Rivers and in parts of the United States and Canada surrounding the Great Lakes and the Saint Lawrence River (*2*,*5*). Hyperendemic foci exist in north-central Wisconsin and western Ontario. Areas of blastomycosis endemicity are based primarily on reports of symptomatic disease and was first described in the late 1930s (*5*). Although the epidemiology of blastomycosis is not as well understood as that of the other mycoses endemic to North America, the incidence seems to be increasing in some states, including Illinois, Indiana, and Wisconsin (*2*).

Cases of canine blastomycosis are often recognized as sentinels for human disease, presumably because dogs’ outdoor exposures are similar to, but potentially more extensive than, those of their human counterparts (*6*). A study in Illinois estimated blastomycosis incidence among dogs to be >8 times that among humans, and during 2001–2007, the annual incidence among dogs increased 50-fold (*7*). In addition to these apparent increases, blastomycosis has also been documented far outside the known disease-endemic areas. One report describes 2 cases probably acquired in the Pacific Northwest: 1 case of ocular blastomycosis in a golden retriever that had never left Washington state and 1 case of disseminated disease in a Rottweiler mix with no history of travel outside of British Columbia (Table 1) (*8*).

**Table 1:** Cases of blastomycosis, coccidioidomycosis, and histoplasmosis acquired outside the traditionally defined mycosis-endemic areas, United States*

**Infection and reference**	**No. cases, species**	**Location**	**Type of infection**	**Method of diagnosis**
Blastomycosis				
(*8*)	1, dog	Central Washington	Ocular	Histopathology and culture
(*9*)	1, human	Oregon	NS	Culture
(*10*)	1, human	North-central Florida	Primary cutaneous	Histopathology
(*11*)	2, human	Western (1 case) and central Nebraska (1 case)	Bone	Culture plus confirmation by DNA probe
(*12*)	2, human	Central Colorado	Pulmonary	Histopathology and culture (1 case), histopathology and culture plus confirmation by DNA probe (1 case)
Coccidioidomycosis				
(*13*)	61, human	Butte County, California	Pulmonary, complications NS	Clinical findings (61 cases), skin test or serology (27 cases)
(*14*)	17, human	Tehama County, California	Pulmonary, complications NS	Clinical findings (17 cases), skin test or serology (10 cases)
(*15*)	10, human	Northeastern Utah	Pulmonary, complications NS	Clinical findings (10 cases), serology (9 cases)
(*16*)	3, human	South-central Washington	1 pulmonary, 1 primary cutaneous, 1 pulmonary → meningitis	Culture and serology
Histoplasmosis				
(*8*)	1, dog	Western Idaho	Disseminated	Histopathology and PCR
(*17*)	1, otter	Kodiak Island, Alaska	Disseminated	Histopathology and PCR
(*18*)	5, cats	California (2 cases), Colorado (2 cases), and New Mexico (1 case)	Disseminated (4 cases), localized (1 case)	Histopathology and PCR (4 cases), culture and PCR (1 cases)
(*19*)	2, cats	San Joaquin Valley, California	Pulmonary (1 case), cutaneous/ocular (1 case)	Cytopathology (1 case), cytopathology and culture plus confirmation by DNA probe (1 case)
(*20*)	2, raccoons	San Francisco, California	Disseminated	Histopathology, culture, and PCR
(*21*)	2, human	California	NS	Skin test and chest x-ray
(*22*)	1, human	California	Endocarditis	Histopathology, PCR, and urine EIA
(*23*)	1, human	Arizona	Gastrointestinal → central nervous system	Histopathology and culture
(*24*)	6, human	Southwestern (3 cases) and eastern (2 cases) Montana; southwestern Idaho (1 case)	Disseminated (3 cases), pulmonary (1 case), unknown (2 cases)	Histopathology (2 cases), culture (2 cases), urine EIA (2 cases)
(*25*)	5, human	Southern Florida	Disseminated	Histopathology
(*26*)	1, human	Northern Florida	Pulmonary	Skin test and serology
(*27*)	15, human	Central New York		Histopathology (10 cases), serology (9 cases)
(*28*)	1, human	Staten Island, New York	Pulmonary	Skin test and serology
(*29*)	5, human	South Bronx, New York	Disseminated	Culture

*EIA, enzyme immunoassay; NS, not specified.

Similarly, several cases of blastomycosis in humans have also occurred far outside the traditionally defined disease-endemic area. A report from 1951 describes disseminated blastomycosis in an agricultural worker in Oregon (*9*). More recently, an immunosuppressed 6-year-old girl was thought to have contracted primary cutaneous blastomycosis in north-central Florida (*10*). Two unrelated cases of osseous blastomycosis of the knee without apparent pulmonary involvement were probably acquired in Nebraska (*11*): the first case was in a previously healthy man from western Nebraska who sustained a knee injury involving farm equipment ≈1 year before diagnosis, and the second case was in a man from central Nebraska with a 1-month history of knee pain but no acute knee injury (*11*). These cases highlight the difficulties associated with diagnosing blastomycosis in patients with no exposures to disease-endemic areas and whose disease manifestations are unusual (*11*). Similarly, 2 other cases in previously healthy adults occurred in Colorado (*12*); each patient had pulmonary disease and had been extensively exposed to soil while excavating prairie dog burrows on the eastern slope of the Rocky Mountains (*12*). De Groote et al. suggest that higher-than-normal rainfall in that region could have contributed to conditions favorable for *Blastomyces* growth and sporulation (*12*).

Moisture is believed to be an influential factor in the growth and dispersal of *Blastomyces*, although the precise ecology of the organism is not well understood, partly because of the difficulties associated with its recovery from the environment (*2*). Previously, blastomycosis was believed to be caused by 1 species, *B. dermatitidis*, but phylogenetic analysis indicates that *B. dermatitidis* is probably 2 species, *B. dermatitidis* and *B. gilchristii* (*30*). Furthermore, *B. gilchristii* is hypothesized to inhabit a specific ecologic niche in areas of hyperendemicity, whereas *B. dermatitidis* may be adapted to a wider range of environmental conditions and distribution throughout North America may be scattered (*30*). Additional research into the genetic, geographic, and clinical differences between these 2 species may contribute to a better understanding of blastomycosis epidemiology, both inside and outside the traditionally defined areas of endemicity.

### Coccidioidomycosis

Coccidioidomycosis is caused by *Coccidioides* spp. and is endemic to the southwestern United States and parts of Mexico and Central and South America. An estimated 60% of *Coccidioides* infections are asymptomatic (*3*,*31*). The remaining 40% of infections most commonly cause an influenza-like illness also known as Valley fever, which is often self-limiting but can result in serious illness, particularly because some cases progress to severe pulmonary or disseminated disease. Prior *Coccidioides* infection usually provides immunity against reinfection, which can be assessed by use of a skin test antigen.

The first complete description of the geographic distribution of coccidioidomycosis was accomplished through large-scale evaluations of coccidioidin sensitivity prevalence. During the mid-1940s to early 1950s, coccidioidin skin tests were performed on ≈110,000 lifetime residents of a single county, most of whom were white men and women 17–21 years of age (*32*). The study identified Arizona, California, Nevada, New Mexico, Utah, and Texas as *Coccicioides*-endemic states; the highest rates of skin test positivity (50%–70%) were in California’s southern San Joaquin Valley and Arizona’s Sonoran Desert (*32*). Point-source coccidioidomycosis outbreaks near the borders of the areas identified by Edwards and Palmer further contributed to what is known about the geographic distribution of *Coccidioides* spp. (*32*). For example, in an outbreak that occurred in 1970, at least 61 archeology students were affected after excavating Native American ruins near Chico, Butte County, California, ≈70 miles north of the recognized disease-endemic area at the time (*13*). In 1972, at least 17 persons were infected during a similar excavation near Red Bluff, Tehama County, California, 20 miles north of the previous outbreak near Chico (*14*). In 2001, another outbreak occurred among 10 workers at an archeological site in Dinosaur National Monument in northeastern Utah, ≈200 miles north of the previously defined disease-endemic area (*15*). These outbreaks support the idea that foci of *Coccidioides* exist outside of the traditional areas of endemicity, yet they are not always represented on maps depicting these areas.

During 2010–2011, three unrelated coccidioidomycosis cases were identified in south-central Washington, far north of the area of known *Coccidioides* endemicity (*16*). Whole-genome sequencing of 1 clinical isolate from the patient and soil isolates recovered from the patient’s location of exposure revealed that the isolates were identical, providing direct evidence that the infection was acquired in Washington (*33*).

Evidence of coccidioidomycosis far outside the areas of known endemicity has also been seen in fossil records. *Coccidioides* spherules were morphologically identified in 2 fossilized, 8,500-year-old bison mandibles recovered from a flood plain in central Nebraska, suggesting that bison had migrated from disease-endemic areas or that *Coccidioides* previously inhabited a different or broader geographic range (*34*).

### Histoplasmosis

Histoplasmosis-endemic areas were also established by using nationwide skin testing to evaluate histoplasmin sensitivity among ≈70,000 white persons, 17–21 years of age, who were lifetime residents of a single county (*35*). The highest proportion of positive reactors (60%–90%) occurred in states bordering the Ohio and Mississippi River valleys; a zone of moderate prevalence (30%–60%) extended outward around the central area for up to 300 miles (*35*). *Histoplasma* spp. seems to be less geographically restricted than *Blastomyces* or *Coccidioides* and is also endemic to parts of central America and various other locations worldwide, such as Africa and Asia. Because *Histoplasma* grows well in soil containing bird or bat droppings, the organism probably exists in microfoci outside of these broadly defined regions (*4*).

*Histoplasma* can infect many animal species and has been found in domestic and wild animals far outside the traditional disease-endemic areas, including disseminated disease in a dog with no history of travel outside of western Idaho (*8*) and in a northern sea otter found in Alaska (*17*). Otters do not migrate, so the authors of that report hypothesized that the infection was acquired from windborne spores or spores carried on the wings, feet, or beaks of migratory birds (*17*). Spores carried in seawater may be another possible explanation (*36*).

Histoplasmosis has also occurred in cats in the putatively non–disease-endemic states of Colorado, New Mexico, and California (*18*,*19*). Multilocus sequence typing of cat tissue samples indicated that the infecting strains of *H. capsulatum* were closely related to but clustered separately from the North American-1 clade (1 of 2 clades common to North America), suggesting that the genetic differences represent either geographic variation among *H. capsulatum* or differences in the strains capable of infecting animals and humans (*18*). Histoplasmosis in other cats near Vacaville, California (G. Thompson, unpub. data), and in two 6-month-old raccoons rescued near San Francisco (*20*) provides further evidence that *Histoplasma* may be established in California.

Among humans, histoplasmosis potentially acquired in California was first described in 1949; in a series of 5 cases of histoplasmosis in children in California, 1 patient was a girl who had never traveled >100 miles from San Francisco and another was a girl who had lived only in California and Wyoming, which is also not a traditional histoplasmosis-endemic area (*21*). Notably, these histoplasmosis diagnoses were based on chest radiograph findings and positive results for histoplasmin skin tests, which can cross-react with *Coccidioides*. A more recent case occurred in the Central Valley of California, in an immunocompetent 87-year-old man who seroconverted during a febrile illness and in whom *Histoplasma* endocarditis later developed (*22*). Another case provides further evidence of histoplasmosis far west of the traditional disease-endemic area; in an immunocompetent woman from Arizona with no history of travel to histoplasmosis-endemic areas, an initial gastrointestinal infection was later followed by development of an intramedullary spinal cord abscess (*23*). Another 6 unrelated cases were reported from Montana (5 cases) and Idaho (1 case); of these 6 patients, 5 had immunocompromising conditions and 3 experienced substantial diagnostic delays, probably because of the low index of suspicion for histoplasmosis in an unusual location (*24*).

Histoplasmosis has also been observed both south and north of the known disease-endemic areas. In a series of 7 cases of disseminated histoplasmosis in HIV/AIDS patients from south Florida, 5 patients had no relevant travel history (*25*). In northern Florida, acute pulmonary histoplasmosis developed in a college student after he had explored a bat-infested cave (*26*). New York is repeatedly described as a non–histoplasmosis-endemic area and is typically not represented on maps depicting histoplasmosis-endemic areas, probably because the skin test surveys by Manos et al. estimated <10% positive reactors for the entire state and <2% positive reactors in certain counties (*35*). However, cases reported from New York date back several decades, including an outbreak beginning in 1978 at a prison in rural central New York; this outbreak was suspected to have been related to removal of accumulations of bird droppings and trees that served as bird roosting sites (*27*). Evidence also exists of histoplasmosis acquisition in urban areas of New York, such as in a previously healthy child from Staten Island with extensive exposure to birds (*28*) and in a series of 5 cases of disseminated histoplasmosis in HIV/AIDS patients from the south Bronx who had no exposures to birds or bats (*29*).

## Discussion

Reasons for the observed occurrences of blastomycosis, coccidioidomycosis, and histoplasmosis outside areas to which they are traditionally classified as endemic are unclear but are probably multifaceted. Because weather and climate affect the growth and distribution of these fungi in these regions, these factors might also contribute to environmental conditions that could support these fungi elsewhere. The role of animal vectors in the life cycles and geographic distribution of these fungi is unknown; rodents have been suggested as possible reservoirs for *Coccidioides*, and birds and bats can carry *Histoplasma*, indicating that they may be capable of introducing microfoci in geographic regions not considered to be endemic (*37*). In contrast, areas in which endemicity seems to be emerging might represent areas of previously unrecognized disease. Despite potential increased clinician awareness, these diseases probably remain underdiagnosed. Documented cases also probably represent the most severe or symptomatic cases, although most cases are unrecognized, and self-resolution is the norm. Fungal infections outside the traditional mycosis-endemic areas clearly represent an emerging public health issue; however, the full scope of the problem remains largely unknown.

Maps of the mycosis-endemic areas are based on outdated and incomplete data and are often reprinted without substantial revisions, even as new data regarding suspected endemicity become available. Although sufficient evidence exists that the original descriptions of these diseases’ distributions are probably not entirely representative of their true range, no comprehensive attempts have been made to systematically reevaluate these estimates. Several potential strategies exist to clarify the true areas of endemicity for these diseases (Table 2). 

**Table 2:** Advantages and disadvantages of potential strategies to refine areas of blastomycosis, coccidioidomycosis, and histoplasmosis endemicity

Strategy	Advantages	Disadvantages
Skin testing	Could cover large geographic areas; is likely to yield results that could be easily compared with early studies of skin test reactivity distribution	Availability, specificity, and cost of reagents may be limiting; may be difficult to identify persons who have no relevant travel history
Expand surveillance for fungal diseases in humans	Provides foundation for more comprehensive surveillance already in place in some states; would provide valuable information about the overall epidemiology of these diseases	Disease reporting can be time- and resource-intensive for state and local health departments; yield for areas of low or no endemicity is potentially low; not likely to capture information on asymptomatic infections; may be difficult to pinpoint location of exposure or rule out reactivation disease in persons who have extensive travel histories
Surveillance for fungal diseases in animals	Animals can be good sentinels for human disease because of potentially more extensive environmental exposures and limited travel	No comprehensive surveillance systems are currently in place; would be time and resource intensive to establish
Improved environmental detection	Detection of fungi in the environment can be a more direct measure of endemicity than disease data; positive results can provide a more definitive link between infection and the environment	Culture-based methods are insensitive; new technologies still in development; is challenging for large geographic areas
Additional ecologic niche modeling	Leads to increased understanding of the fundamental niche for these fungi and locations where human or animal exposures could occur	Model validity relies on the quality of reported locations of human and animal diseases, environmental sampling results, or both

First, the distributions of histoplasmosis and coccidioidomycosis were historically defined by large-scale skin test surveys, and it is reasonable to assume that a similar method could be used to further refine our understanding of these areas. Skin test reagents to detect prior exposure to *Histoplasma* and *Coccidioides* have been unavailable in the United States for more than a decade, but a reformulated spherule-derived skin test antigen (Spherusol; AllerMed, San Diego, CA, USA) to detect delayed-type hypersensitivity to *Coccidioides* was recently approved by the Food and Drug Administration. Although access to skin testing may enable reduction of potential exposure for nonimmune patients, widespread skin test surveys to reevaluate areas of endemicity may be difficult to implement because of cost and concerns about reagent specificities.

Second, improved disease surveillance and mandatory case reporting could improve detection of infections in persons not exposed to areas of known endemicity and would contribute to a more comprehensive epidemiologic understanding of these infections in general. One of the current challenges with state-based reporting methods is that these diseases are typically reportable only in states within the traditionally defined areas of endemicity (and are sometimes not reportable even in states with known endemicity); thus, infections acquired in unusual geographic locations probably go undocumented. In addition, identifying the location of exposure for persons with histories of travel to places where these diseases are most common may be difficult. Because exposures of animals are theoretically similar to those of humans but animals travel less, animals can be good sentinels for human disease; however, no comprehensive surveillance methods exist to monitor fungal diseases in animals. Epidemiologic surveillance for blastomycosis, coccidioidomycosis, and histoplasmosis in humans and animals fundamentally relies on accurate diagnosis. The challenges associated with diagnosing these infections are well recognized; sensitivity of histopathology and cytology is low, and cross-reactions between the endemic mycoses are a particular concern with serologic testing (*2*–*4*). Our review is subject to the same limitations as those of the original reports regarding diagnostic methods for non–culture-confirmed cases.

Third, because detection of pathogenic fungi in environmental samples is a more direct measure of whether a given location can support the fungus than are data about the occurrence of disease, environmental studies could be helpful for further characterizing the areas of endemicity. Testing of environmental samples for *Coccidioides*, *Histoplasma*, or *Blastomyces* has traditionally relied on culture methods and animal inoculation, which are associated with low sensitivity and which are labor and resource intensive; however, molecular methods such as PCR are promising (*33*). As these technologies advance and become more widely used, they may serve as valuable tools to help determine sources of infection in environmental material and air. 

Last, ecologic niche modeling has been beneficial for understanding the probable distribution and environmental conditions favorable for *Blastomyces* (*38*) and *Coccidioides* (*39*). Better environmental detection methods could help refine the inputs for these types of models, thereby improving their predictive abilities.

The concept of endemicity of fungal diseases is well established and has proven useful, but it may, in some instances, be potentially misleading. An “endemic disease” is one “occurring frequently in a particular region or population” (*40*). The dichotomy of endemic and nonendemic diseases or endemicity or nonendemicitiy in geographic areas may not be fully adequate to capture the nuances of fungal disease epidemiology; the seemingly increasing frequency of acquisition of fungal infections in areas well removed from those known to be endemic suggests that consideration should be given to discarding the term “nonendemic” in favor of “not known to be endemic” (Figure). Furthermore, even within a region of known endemicity, there may be areas in which the pathogen seems to be absent, as reflected in a lack of recognition of locally acquired infections. In contrast, some areas within a region of endemicity may be regions of hyperendemicity, contributing large numbers of cases, and others may be regions of hypoendemicity. These conceptual issues pertain not only to space, but also to time; hyperendemicity in some areas may be seasonal or endemicity only transient. Approaching the notion of endemicity for fungal diseases with a more nuanced and dynamic view has both epidemiologic and clinical value. Future work defining fungal disease endemicity should use a classification such as this.

**Figure:**
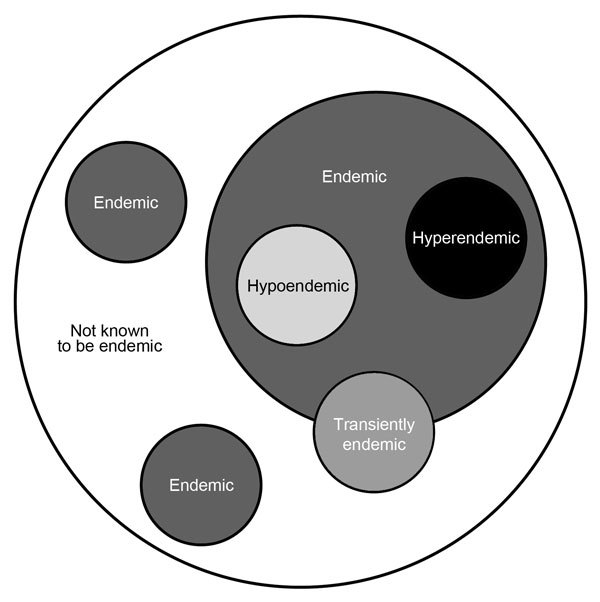
Proposed classification for endemicity of fungal infections. This schematic depicts the range of endemicity of fungal infections and discards the notion of “nonendemic,” replacing it with “not known to be endemic,” accounting for new areas of infection acquisition. It also accounts for the variability in the intensity of endemicity and indicates that the presence of a fungus in the environment may be transient as the result of environmental influences.

Because primary prevention of these infections is extremely difficult, early diagnosis and treatment are particularly beneficial and may contribute to improved outcomes. The nonspecific symptoms of blastomycosis, coccidioidomycosis, and histoplasmosis are often clinically indistinguishable from those of other community-acquired respiratory illnesses. Mild or self-resolving cases frequently go undetected, and diagnoses may be missed or delayed, especially in settings where these diseases are uncommon. As a result, diagnoses in unexpected geographic locations probably represent the most severe cases. Therefore, clinicians should not exclude the possibility of these infections in patients who have not been exposed to known areas of endemicity. 

The clinical and public health challenges associated with these diseases are not limited to the United States; the distribution of *Blastomyces* and *Histoplasma* extends into Canada, and histoplasmosis and coccidioidomycosis comprise a substantial burden of disease in parts of Central and South America. Further ecologic and epidemiologic studies, including revision of the geographic distribution, are needed to provide a better understanding the public health implications of these fungal diseases in the United States and elsewhere around the world.
